# Allergen-Specific IgG Antibodies Purified from Mite-Allergic Patients Sera Block the IgE Recognition of *Dermatophagoides pteronyssinus* Antigens: An *In Vitro* Study

**DOI:** 10.1155/2013/657424

**Published:** 2013-08-28

**Authors:** Isabella Lima Siman, Lais Martins de Aquino, Leandro Hideki Ynoue, Juliana Silva Miranda, Ana Claudia Arantes Marquez Pajuaba, Jair Pereira Cunha-Júnior, Deise Aparecida Oliveira Silva, Ernesto Akio Taketomi

**Affiliations:** Laboratory of Allergy and Clinical Immunology, Institute of Biomedical Sciences, Federal University of Uberlândia, Avenida Pará 1720, Bloco 4 C, Campus Umuarama, 38400-902 Uberlândia, MG, Brazil

## Abstract

One of the purposes of specific immunotherapy (SIT) is to modulate humoral immune response against allergens with significant increases in allergen-specific IgG levels, commonly associated with blocking activity. The present study investigated *in vitro* blocking activity of allergen-specific IgG antibodies on IgE reactivity to *Dermatophagoides pteronyssinus* (Dpt) in sera from atopic patients. Dpt-specific IgG antibodies were purified by ammonium sulfate precipitation followed by protein-G affinity chromatography. Purity was checked by SDS-PAGE and immunoreactivity by slot-blot and immunoblot assays. The blocking activity was evaluated by inhibition ELISA. The electrophoretic profile of the ammonium sulfate precipitated fraction showed strongly stained bands in ligand fraction after chromatography, compatible with molecular weight of human whole IgG molecule. The purity degree was confirmed by detecting strong immunoreactivity to IgG, negligible to IgA, and no reactivity to IgE and IgM. Dpt-specific IgG fraction was capable of significantly reducing levels of IgE anti-Dpt, resulting in 35%–51% inhibition of IgE reactivity to Dpt in atopic patients sera. This study showed that allergen-specific IgG antibodies purified from mite-allergic patients sera block the IgE recognition of *Dermatophagoides pteronyssinus* antigens. This approach reinforces that intermittent measurement of serum allergen-specific IgG antibodies will be an important objective laboratorial parameter that will help specialists to follow their patients under SIT.

## 1. Introduction

Allergic diseases are considered one of the major health problems worldwide and constitute a breakdown in the immune tolerance against natural exposure to environmental antigens [1]. Among them, the house dust mites (HDMs) from the family Pyroglyphidae, mostly *Dermatophagoides pteronyssinus*, play an important role to induce respiratory allergic diseases, particularly asthma and allergic rhinitis, in genetically predisposed individuals [2, 3]. Recent data from World Allergy Organization (WAO) show that the prevalence of allergic diseases has occurred in 30%–40% of the world population, of which 400 million suffer from allergic rhinitis [4]. The immediate symptoms of allergic rhinitis are caused by allergen-induced crosslinking of mast cell-bound IgE antibodies and release of inflammatory mediators as histamine and leukotrienes [5].

Several studies have been performed focusing on the development of new treatments beyond the pharmacotherapy already established, aiming to relieve the symptoms caused by exacerbated responses of the organism against allergens [6]. Allergen-specific immunotherapy (SIT) is the main treatment used for allergy and involves mechanisms that include the production of blocking antibodies, the shifts toward Th1 response, and tolerance induction [7, 8]. IgG antibodies induced by SIT may act as blocking agents by competing with IgE for allergen binding, inhibiting the activation of IgE-dependent mast cells and basophils and reducing IgE-mediated allergic inflammation [5, 9]. Previous studies have attributed to IgG subclasses, particularly IgG4, a protective activity, by acting like an inhibition factor of IgE-mediated hypersensitivity reaction after long-time antigen exposure. Consequently, IgG4 antibodies might neutralize allergens or block IgE binding to allergens, attenuating thereby the allergic reaction [10, 11]. Even though SIT induces high levels of specific IgG1 and IgG4 [12], the blocking capacity of these antibody classes remains to be clarified. Thus, the knowledge about the role and mechanisms of blocking activity of these antibodies can facilitate the progress and development of new techniques for SIT [13]. 

Considering that a successful SIT correlates with decreasing of clinical symptoms and increasing of allergen-specific IgG antibody levels, the aim of this study was to investigate *in vitro* the blocking capability of specific IgG antibodies purified from mite-allergic patients sera on the IgE reactivity to *D. pteronyssinus*.

## 2. Materials and Methods

### 2.1. Subjects and Skin Prick Test

Thirty-six patients, male and female, aged 18 to 60, with clinical history of allergic rhinitis (atopic group) were recruited from the Laboratory of Allergy and Clinical Immunology, Federal University of Uberlandia, Uberlandia, MG, Brazil. As inclusion criteria, patients should have positive skin prick test (SPT) to at least *Dermatophagoides pteronyssinus *(Dpt) allergen extract from a panel of standardized aeroallergens (FDA Allergenic Ltda, Rio de Janeiro, RJ, Brazil) as follows: house dust mites (*D. pteronyssinus*, *D. farinae, and Blomia tropicalis*); cockroaches (*Blattella germanica* and *Periplaneta americana*); mold (*Alternaria alternata*); and pet danders (*Felis domesticus* and *Canis familiaris*). The exclusion criteria were positive results in rheumatoid factor assay (Bio Látex FR, Bioclin, Belo Horizonte, MG, Brazil), the use of antihistamines in the previous week to the skin test, and previous or current immunotherapy. 

Fifteen volunteers, healthy subjects, male and female, aged 18 to 60, were selected based on the absence of clinical history or symptoms of allergic rhinitis and negative SPT to a panel of standardized aeroallergens (nonatopic group). In parallel, blood samples (10 mL) were collected from all individuals, and the serum was stored at −20°C until serological assays. 

The study was approved by the Ethics Committee in Human Research of the Federal University of Uberlandia, and written informed consent was obtained from all participants.

### 2.2. Measurement of *D. pteronyssinus*-Specific IgE, IgG1, and IgG4

All serum samples were assessed by enzyme linked immunosorbent assay (ELISA) for measuring levels of IgE, IgG1, and IgG4 to Dpt as previously described [14, 15], with some modifications. Briefly, microtiter plates were coated with Dpt extract (2 *μ*g/well; Hollister-Stier Laboratories, Spokane, WA, USA), blocked with phosphate-buffered saline (PBS, pH 7.2) containing 0.05% Tween 20 and 1% bovine serum albumin (PBS-T-BSA) for IgE and PBS-T-BSA 0.1% for IgG1 and IgG4, and then incubated with serum samples diluted 1 : 2 (IgE), 1 : 10 (IgG1), or 1 : 5 (IgG4) for 2 hours at 37°C. After washing, plates were incubated with biotinylated secondary antibodies as anti-human IgE (1 : 1,000; Kirkegaard and Perry Laboratories Inc. (KPL), Gaithersburg, MD, USA), anti-human IgG1 (1 : 3,000; Sigma Chemical Co., St Louis, MO, USA), or anti-human IgG4 (1 : 1,000; Sigma) for 1 hour at 37°C and subsequently with streptavidin peroxidase (1 : 1,000; Sigma). The assay was developed with ABTS-peroxidase substrate system (KPL), and optical density (OD) values were determined at 405 nm. Antibody levels were expressed as ELISA index (EI) according to the following formula: EI = OD test sample/cutoff, where the cutoff was established as the mean OD value of negative control sera plus 3 standard deviations. EI values >1.2 were considered to be positive in order to exclude borderline reactivity values close to EI = 1.0.

### 2.3. Serum Pools and Salting-Out Precipitation

Five serum samples of each patient group were selected to constitute the Dpt-specific (atopic) and nonspecific (nonatopic) serum pools. The selection criteria were based on the highest (EI > 2.0) and lowest (EI < 1.0) values of reactivity to both IgG1 and IgG4 to Dpt allergen in atopic and nonatopic groups, respectively. Thus, these criteria would allow obtaining a maximal diversity in antigen/epitope recognition, according to different seroreactivity profiles and intensity of reaction observed in immunoenzymatic assays, favouring the identification of biological phenomena rather than individual immune responses. Initially, the serum albumin from specific and nonspecific pools was partially depleted by salting-out precipitation using 40% ammonium sulfate [16]. The supernatant (S 40%) and precipitated (P 40%) fractions obtained were dialyzed and concentrated by using Amicon system (Millipore, Billerica, MA, USA) and further analyzed in polyacrylamide gel electrophoresis with sodium dodecyl sulfate (SDS-PAGE) on 8% gels under nonreducing conditions. Samples were solubilized in sample buffer, boiled at 96°C for 5 min, and applied to the gel in parallel with molecular weight markers (BenchMark Protein Ladder, Invitrogen, Carlsbad, CA, USA). Protein profile was visualized with blue silver staining [17]. 

### 2.4. Slot-Blot Assays

Nitrocellulose membranes (0.45 *µ*m; Bio-Rad Laboratories Inc., Hercules, CA, USA) were coated with serum pools, S 40% and P 40% fractions, and bovine serum albumin (BSA) as negative control, using the Mini Protean II Multiscreen Apparatus (Bio-Rad). After blocking with PBS-T plus 5% skim milk, membranes were incubated with detection antibodies labeled with peroxidase (for IgG diluted 1 : 5,000 and IgM diluted 1 : 5,000; Calbiochem Merck, Darmstadt, Germany), biotin (for IgA diluted 1 : 10,000; Sigma), and monoclonal antibody to IgE (1 : 1,000; Sigma). After incubation with streptavidin peroxidase (1 : 1,000; Sigma) or anti-mouse IgG/peroxidase (1 : 1,000; Oncogene Science, Cambridge, MA, USA), when appropriate, the membranes were revealed with DAB tablets (Sigma). Bands were analyzed using the ImageJ 1.46 software (National Institute of Mental Health, Bethesda, MA, USA).

### 2.5. Purification of Total IgG by Affinity Chromatography and Immunoblots

The P 40% fractions of each specific and nonspecific serum pool were loaded into affinity chromatography columns (Pierce Protein G Agarose, Thermo Fisher Scientific Inc., Rockford, IL, USA), previously equilibrated with binding buffer (0.02 M phosphate buffer, pH 8.0). Samples were diluted 1 : 1 in binding buffer, applied to the column, and washed with at least 10 volumes of binding buffer. Total IgG was eluted in elution buffer (0.1 M glycine, pH 2.6), and 1 mL fractions were collected. After pH neutralization with 1 M Tris-HCl, pH 9.0, absorbance was read at 280 nm. Values of absorbance and pH of each fraction prior to neutralization were used to build the chromatographic profile. 

The purity of total IgG obtained from the affinity chromatography was checked using SDS-PAGE on 8% gels as described above. To confirm the immunoreactivity of the eluted fractions, immunoblots were performed for detection of IgG, IgA, IgE, and IgM antibodies. Briefly, the fractions previously separated on 8% SDS-PAGE were electrotransferred onto nitrocellulose membranes, and blotting efficiency was validated by reversible Ponceau S staining. Membranes were blocked with 5% skim milk in PBS-T. After blocking, blots were incubated with the respective detection antibodies to IgG, IgA, IgE, and IgM as described in slot-blot assays and revealed with DAB. 

After monitoring the eluted fractions by 8% SDS-PAGE, the samples containing IgG were pooled, dialyzed, and concentrated against PBS by using Amicon system. Protein concentration was determined using the Lowry method [18]. 

### 2.6. Determination of Optimal Concentrations of Specific and Nonspecific IgG Fractions

An indirect ELISA was carried out to determine the optimal concentrations of specific and nonspecific IgG fractions through detection of IgG1 and IgG4 anti-Dpt as well as residual specific IgE as previously described [14, 15], with modifications. Briefly, plates were coated with Dpt (2 *μ*g/well), blocked with PBS-T-BSA 0.1%, and subsequently incubated with specific and nonspecific IgG fractions diluted from 40 to 2.5 *μ*g/well. Subsequent steps were similar to the ELISA for detection of Dpt-specific IgE, IgG1, and IgG4 as described above.

### 2.7. Inhibition ELISA

To evaluate the blocking activity of the Dpt-specific IgG fractions on IgE reactivity to Dpt allergen, an inhibition ELISA was developed by using three serum pools of atopic patients with different positivity for Dpt-specific antibody classes as follows: pool I (IgE+, IgG1+, and IgG4–); pool II (IgE+, IgG1–, and IgG4+); and pool III (IgE+, IgG1+, and IgG4+). Briefly, plates were coated with Dpt (2 *μ*g/well), blocked with PBS-T-BSA, and incubated with the optimal concentration of specific or nonspecific IgG fractions for 1 h at 37°C. Then, the serum pools from atopic patients were diluted 1 : 2, incubated for 1 h at 37°C, and followed by incubation with biotinylated anti-human IgE (1 : 1,000; KPL). Subsequent steps were similar to the ELISA for detection of IgE anti-Dpt as described above. Results were reported as absorbance values at 405 nm and inhibition percentage as follows: % inhibition = 1 − (DO inhibited/DO uninhibited) × 100 [19].

### 2.8. Statistical Analysis

Statistical analysis was performed using GraphPad Prism version 5.0 (GraphPad Software Inc.). Comparison between levels of IgE, IgG1, and IgG4 antibodies to Dpt within the groups was analyzed by the Mann-Whitney test. Differences in slot-blot data were determined by Student's *t*-test. Differences between the groups were analyzed by one-way ANOVA using the Bonferroni posttest (IgE reactivity and inhibition ELISA). Correlation between the levels of antibody classes was analyzed by the Spearman correlation test. Values of *P* < 0.05 were considered statistically significant. 

## 3. Results

The demographic and clinical characteristics of the study subjects are shown in Table 1. All patients from the atopic group had clinical history of allergic rhinitis related to HDMs exposure and positive SPT to aeroallergen extracts, with higher concomitant sensitization to HDMs, *D. pteronyssinus, *and *D. farinae *(97%) than to *Blomia tropicalis *(64%) and to other aeroallergens (<54%) (*P* < 0.0001). The atopic and nonatopic groups were comparable regarding the sex and age. 

**Figure 1:**
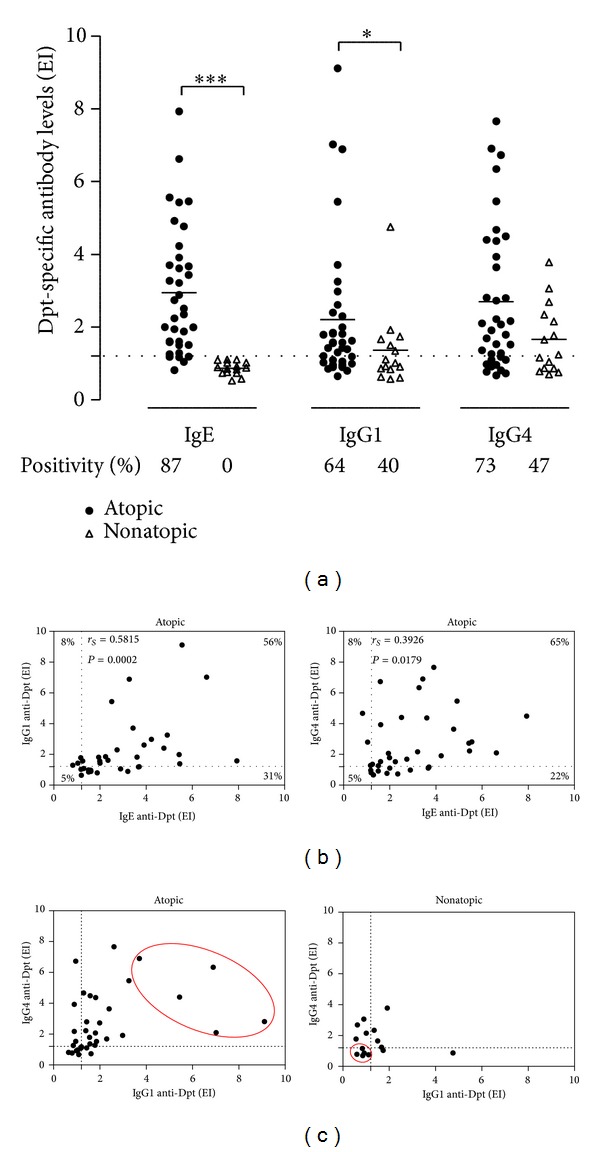
(a) Levels of IgE, IgG1, and IgG4 antibodies to* Dermatophagoides pteronyssinus* (Dpt) allergen extract in serum samples from atopic and nonatopic patients. Data are expressed in ELISA index (EI), and mean is indicated by horizontal bars. The dashed line indicates the cutoff of the reaction (EI > 1.2). Percentages of positive samples are also indicated. Statistically significant differences were determined by the Mann-Whitney test (*P < 0.05; ***P < 0.0001). (b) Correlation between levels of Dpt-specific IgE versus IgG1 and IgE versus IgG4 anti-Dpt in serum samples from atopic patients. Percentages of double positive, double negative, or single positive for each antibody class are indicated in the correspondent corners. Spearman's correlation coefficient and statistical significance are also indicated. (c)**  **Comparison between levels of IgG1 and IgG4 anti-Dpt in serum samples from atopic and non-atopic patients. Five serum samples (red ellipses) of each patient group were selected to constitute the specific (atopic) and nonspecific (nonatopic) total IgG pools. The dashed lines indicate the cutoff of the reaction (EI > 1.2).

Levels of IgE to *D. pteronyssinus* were higher in atopic patients than in nonatopic subjects (*P* < 0.0001; Figure 1(a)), with 87% of positivity in atopics and no positivity in nonatopics. Likewise, levels of IgG1 anti-Dpt were higher in atopics than nonatopics (*P* < 0.05), although the positivity was similar between groups. In contrast, levels and positivity of IgG4 anti-Dpt were similar between the groups. Significant positive correlations were found between Dpt-specific IgE and IgG1 (*r*_*S*_ = 0.5815; *P* = 0.0002) or IgG4 (*r*_*S*_ = 0.3926; *P* = 0.0179), with slightly higher number of double-positive patients for IgE and IgG4 (65%) than for IgE and IgG1 anti-Dpt (56%) (Figure 1(b)).

To select the serum samples with the highest and lowest concomitant IgG1 and IgG4 reactivity within the atopic and nonatopic groups, respectively, levels of IgG1 and IgG4 were compared as shown in Figure 1(c). Five serum samples were selected within each group and pooled to constitute the Dpt-specific and nonspecific serum pools, respectively. The Dpt-specific IgE, IgG1, and IgG4 reactivity profiles in each serum pool revealed mean EI values above 4.2 for the three antibody classes in the atopic group and below 1.0 in the nonatopic group (Table 2). 

**Figure 2:**
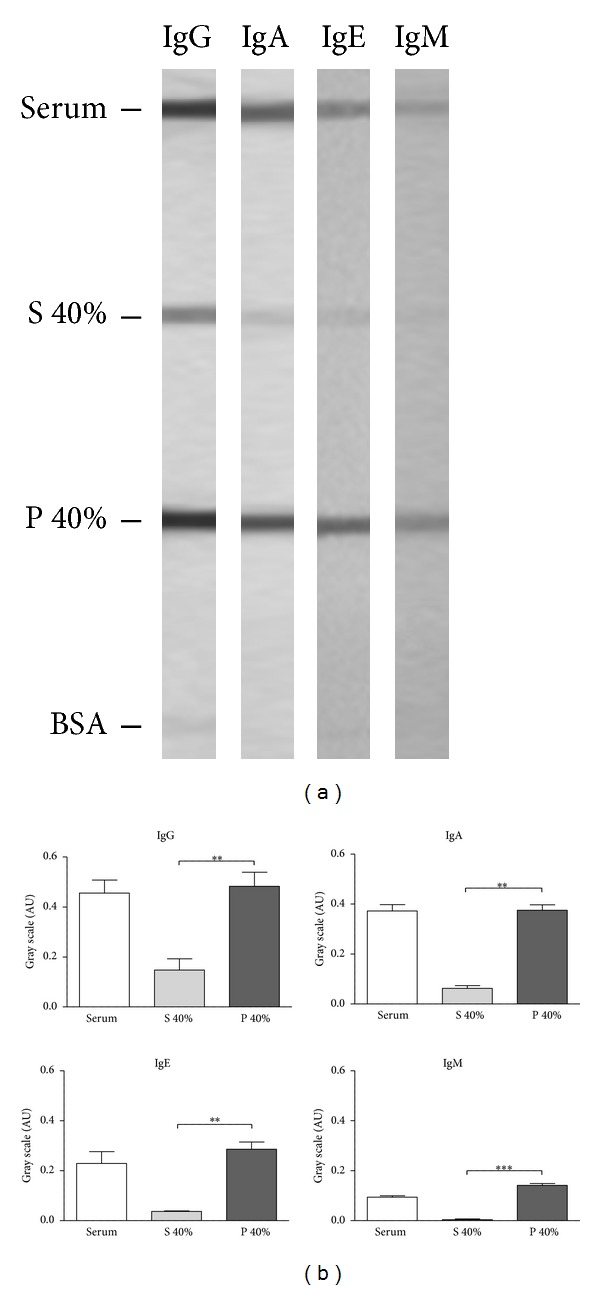
(a) Slot-blots showing reactivity for IgE, IgA, IgG, and IgM in the serum, supernatant (S 40%), and precipitated (P 40%) fractions obtained from precipitation of the serum with 40% ammonium sulfate solution, and bovine serum albumin (BSA) as irrelevant protein. (b) Slot-blot data analysis by measuring the intensity of the bands in gray scale and expressed in arbitrary units (AU). Statistically significant differences were determined by Student's *t* test (**P < 0.01; **P < 0.001).

Total human IgG purification was performed in two steps. Firstly, Dpt-specific and nonspecific serum pools were partly purified by 40% ammonium sulfate precipitation, obtaining the S 40% and P 40% fractions. The immunoglobulin profile in these fractions was verified by slot-blot, showing that all analyzed classes (IgG, IgA, IgE, and IgM) were more concentrated in P 40% than S 40% fractions (*P* < 0.01) as shown in Figures 2(a)–2(b). 

**Figure 3:**
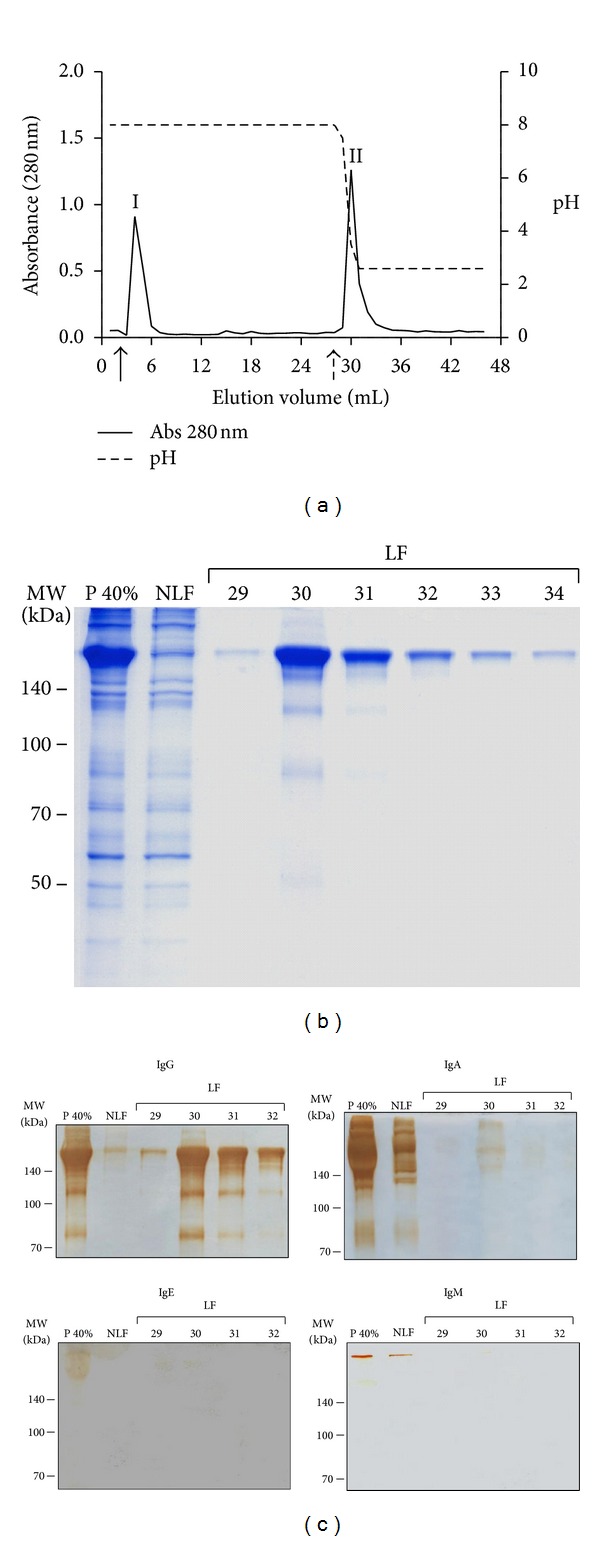
(a) Representative chromatogram of the total human IgG purification by affinity chromatography in protein G-agarose obtained from the 40% ammonium sulfate precipitated fraction (P 40%) of a serum pool. I—elution peak of P 40% nonligand fraction after washing with 0.02 M phosphate buffer pH 8.0 (black arrow); II—elution peak of P 40% ligand fraction after washing with 0.1 M glycine buffer pH 2.6 (dashed arrow). Data are expressed in absorbance (280 nm). Elution volume consisted of 1 mL in each tube. Values of pH were also measured in each elution tube. (b) Electrophoretic profile in SDS-PAGE 8% stained with blue silver. NLF—nonligand fraction, LF—ligand fraction, corresponding to the tubes 29–34. Markers of molecular weight (MW) are indicated on the left in kilodaltons (kDa). (c) Immunoblots for detection of IgG, IgA, IgE, and IgM in the serum fractions after purification in protein G-Agarose as shown in (b). Bands were revealed with DAB as described in Methods.

Secondly, the P 40% fractions of each serum pool were loaded into protein G-agarose column, and a representative chromatogram is illustrated in Figure 3(a). The peak I (tubes 3 to 6) was obtained during washing with binding buffer, representing the nonligand fraction (NLF). The peak II (tubes 29 to 34) was obtained after elution buffer, corresponding to the ligand fraction (LF). To check the purification of these fractions, SDS-PAGE 8% was performed. A representative electrophoretic profile shows strongly stained bands around 160 kDa in the LF fractions, compatible with the molecular weight of whole IgG molecules, including the high and light chains (Figure 3(b)). The immunoreactivity of these LF fractions was verified by immunoblots, showing a strong reactivity to IgG, whereas IgA reactivity was negligible, and no reactivity was detected to IgE and IgM antibodies (Figure 3(c)).

**Figure 4:**
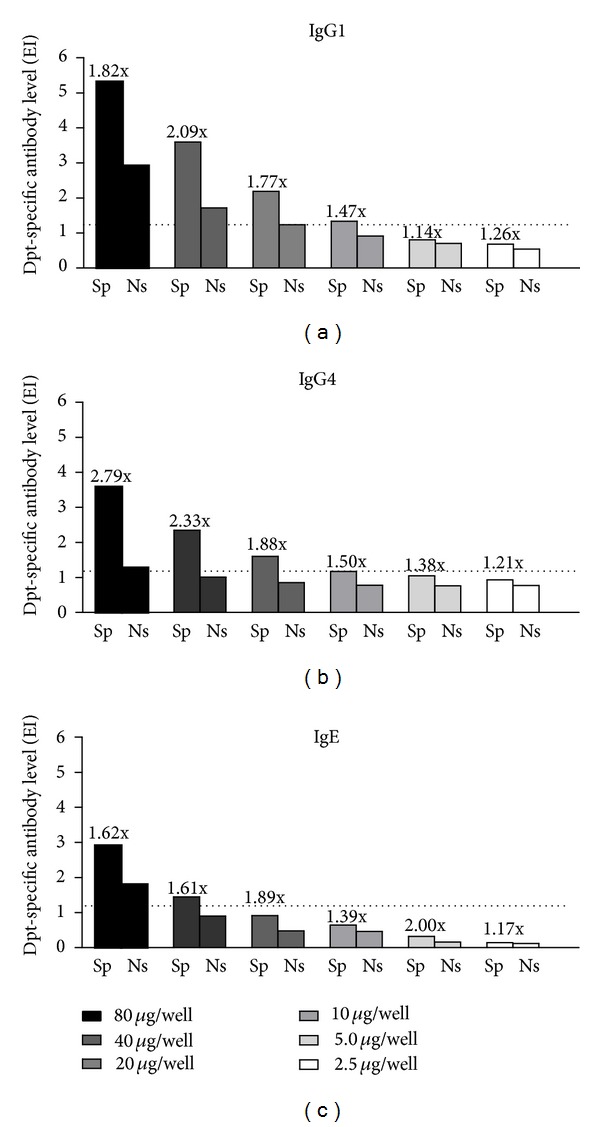
Levels of IgG1 (a), IgG4 (b), and IgE (c) antibodies to *Dermatophagoides pteronyssinus *(Dpt) allergen extract in the specific (Sp) and nonspecific (Ns) purified IgG fractions obtained from atopic and nonatopic patients, respectively, determined by ELISA. Purified IgG fractions were titrated at two-fold dilutions from 80 to 2.5 *µ*g/well, and data are expressed in ELISA index (EI). The dashed lines indicate the cutoff of the reaction (EI > 1.2). The values indicating the Sp/Ns ratio for each antibody class and analyzed concentration are also indicated.

To determine the optimal concentration of Dpt-specific and nonspecific IgG fractions to be used in inhibition ELISA, an indirect ELISA was performed to detect levels of IgG1 and IgG4 anti-Dpt in these fractions. As shown in Figures 4(a)–4(c), the best distinction between the two fractions was found when 40 *μ*g/well was used, considering the ratio of reactivity between specific and nonspecific fractions as well as the cutoff value for each reaction. However, we could not determine the saturating concentration to occupy all the sites because the reaction background generated by nonspecific IgG fractions also increased considerably at 80 *μ*g/well for the three antibody classes, particularly for IgG1 and IgE. It is noteworthy that IgE reactivity detected in both specific and nonspecific IgG fractions at 40 *μ*g/well was borderline or below the cutoff (Figure 4(c)). For these reasons, the concentration of 40 *μ*g/well was chosen for further experiments. To verify which is the predominant antibody subclass in the purified specific IgG fraction at 40 *μ*g/well, the IgG1/IgG4 ratio was calculated (IgG1/IgG4 = 1.53), showing that the predominant antibody subclass in purified specific IgG fractions was IgG1, as expected.

**Figure 5:**
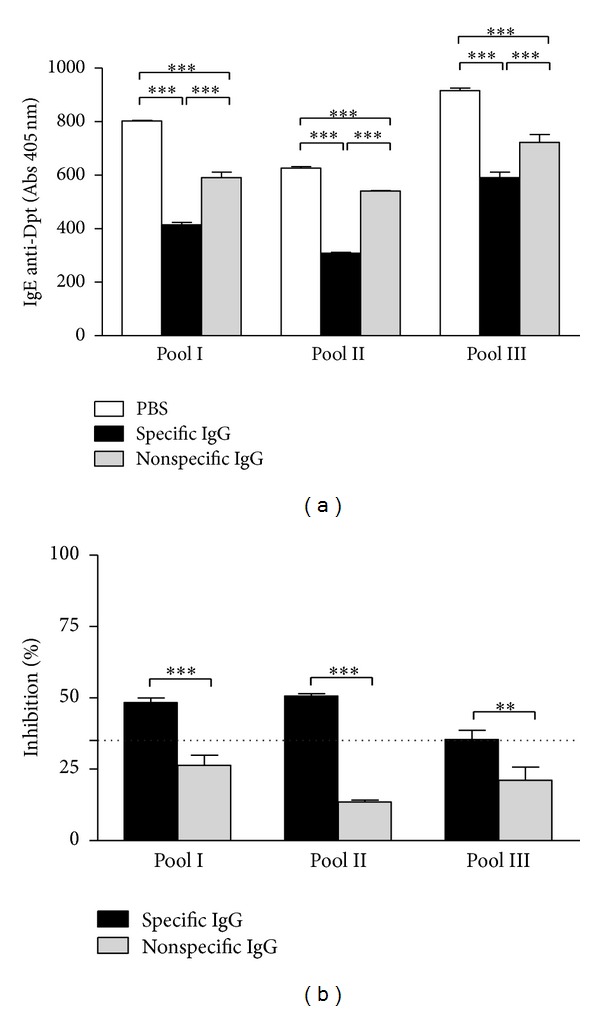
Inhibition ELISA results showing the blocking capacity of specific and nonspecific purified IgG antibodies for IgE reactivity to *Dermatophagoides pteronyssinus *(Dpt) allergen extract in serum pools of atopic patients. Three serum pools (I, II, and III) with different positivity for antibody classes were used as follows: pool I (IgE+, IgG1+, and IgG4−); pool II (IgE+, IgG1−, and IgG4+); and pool III (IgE+, IgG1+, and IgG4+). (a) Levels of IgE anti-Dpt expressed in absorbance (405 nm). Statistically significant differences were determined by one-way ANOVA and the Bonferroni posttest (***P < 0.0001). (b) Percentage of inhibition of IgE binding by blocking specific and nonspecific IgG antibodies in three serum pools of atopic patients. The dashed line indicates a threshold inhibition value of 35%. Statistically significant differences were determined by Student's *t*-test (**P < 0.01; ***P < 0.0001).

Next, IgE reactivity to Dpt in the presence or absence of specific or nonspecific IgG fractions was determined by inhibition ELISA using three serum pools (I, II, and III) of atopic patients selected with basis on the positivity for the three antibody classes: pool I (IgE+, IgG1+, and IgG4−); pool II (IgE+, IgG1−, and IgG4+); and pool III (IgE+, IgG1+, and IgG4+) (Table 3). For the three serum pools tested, the presence of specific IgG fraction significantly reduced IgE reactivity to Dpt when compared to PBS (*P* < 0.0001) (Figure 5(a)). As shown in Table 3, the levels of Dpt-specific IgE detected in the pool II were lower than those found in the pools I and III. Thus, the reactivity of PBS control in the pool II was lower than in pools I and III, but with no significant differences between them (Figure 5(a)). A similar effect of IgE reduction was observed in the presence of nonspecific IgG fractions (*P* < 0.0001). However, the IgE reactivity was significantly lower in the presence of specific fractions than in nonspecific IgG fractions (*P* < 0.0001). When the percentage of inhibition for IgE reactivity was evaluated, the specific IgG fractions were able to inhibit ≥50% IgE reactivity for the pools I and II, and above 35% inhibition for the pool III (Figure 5(b)). 

## 4. Discussion

It is known that the final purpose of allergen-specific immunotherapy is to modulate the immunological profile against allergens and that the benefits achieved are long-lasting even though the therapy is discontinued [20]. Increases in allergen-specific IgG1 and IgG4 levels are associated with blocking activity by preventing IgE binding to the allergen and consequently leading to a reduction of the allergic inflammatory response [10–12]. 

The characterization, quantification, and evaluation of the blocking capability of IgG antibodies on the allergen-IgE interaction using *in vitro* tests represent a main tool to elucidate the role of different types of immunoglobulins in the allergic response. In the present study, this approach was performed initially through purification of Dpt-specific IgG fractions from serum pools of mite-allergic patients and subsequently, evaluation of its blocking capability on the IgE reactivity by inhibition ELISA assays. 

The recruitment of participants for the atopic group of this study was based on clinical history of allergic rhinitis and positive SPT to at least Dpt allergen extract. A previous study in patients of the Triângulo Mineiro region, Brazil [21], showed high positivity percentages to HDM extracts, underlining *D. pteronyssinus* and *D. farinae* as relevant sensitizing agents in this region. IgE levels and seropositivity in atopic patients found in this study resembled those found in our previous work evaluating the levels of IgE, IgA, and IgG4 antibodies to *D. pteronyssinus* and to its major allergens, Der p1 and Der p2, in samples of serum and saliva from allergic and nonallergic children [14]. Although the mean levels of IgG4 to Dpt were higher in atopic than in nonatopic subjects of the present study, this difference was not statistically significant as that found in our previous studies with* D. pteronyssinus *[14] and *Blomia tropicalis* [22], reinforcing that antigens that induce IgE antibodies are also good inducers of IgG4 antibodies. These findings were supported by significant positive correlation found between Dpt-specific IgE and IgG4 antibodies in atopic patients. Likewise, levels of Dpt-specific IgG1 antibodies were higher in atopic than in nonatopic subjects similarly to our findings of IgG1 anti-*B. tropicalis* [22], supporting that IgG1 antibodies might be more closely related to allergen exposure. Also, the significant positive correlation found between Dpt-specific IgE and IgG1 antibodies reinforces these data. There are ongoing discussions on whether IgG4 is a blocking or an anaphylactic antibody and whether IgG1 is associated with exposure and protective role [23, 24].

Considering the highest and lowest concomitant IgG1 and IgG4 reactivities to Dpt in atopic and nonatopic patients, respectively, serum samples were selected to obtain the specific and nonspecific serum pools. These serum pools were partially purified by salting-out precipitation using 40% ammonium sulfate and then submitted to affinity chromatography to get the specific and nonspecific IgG fractions. These techniques of immunoglobulin purification have been widely used with high quality and integrity of recovered antibodies [25, 26]. The use of the ammonium sulfate precipitation allows obtaining high immunoglobulin concentration with considerable level of purity and no damage to its functional activity. The electrophoretic profile after 40% ammonium sulfate precipitation of the specific and nonspecific serum pools showed an enrichment of high molecular weight proteins in the precipitated fraction and a broad depletion of human serum albumin, consistently with the literature data [27]. In addition, all analyzed antibody classes were more concentrated in the precipitated fractions. Using this technique, we were able to optimize the protein G-agarose affinity chromatography, avoiding interferences from other serum proteins, especially serum albumin. 

The electrophoretic profile after affinity chromatography showed strongly stained bands in the ligand fractions, compatible with the molecular weight of the human whole IgG molecule. Although other bands were visible, they can be considered degradation products since they were stained in immunoblot assays for detection of total IgG. The purification was considered successful because a strong reactivity to IgG was detected, whereas IgA reactivity was negligible, and no reactivity was found for IgE and IgM. Although recent studies have been looking for new clarifications concerning the role of IgD in regulation of immune system [28, 29], we did not perform immunoblots for IgD since its serum concentration is despicable when compared with the other immunoglobulins [30]. 

It is well known that SIT leads to an increase in the allergen-specific IgG production [31–33], with the ability to block the IgE-allergen interaction as well as interaction of these complexes with B cells and basophil activation [34]. Several studies have correlated high IgG serum levels, especially IgG1 and IgG4, with clinical responses of patients after SIT [11, 35, 36]. The association of IgG4 with protective activity is related to its function as a blocking antibody or a marker of tolerance induction, resulting in a decreased sensitivity of T cells and consequently in a suppression of the late-phase reactions [37]. As a marker of tolerance induction, IgG4 antibody measurements may be particularly valuable in follow-up studies, where a considerable increase in IgG4 levels can be a strong indicator of the activation of tolerance-inducing mechanisms [38]. 

In the present study, we obtained Dpt-specific IgG fractions purified from serum pools of atopic patients that were not under any immunotherapy. In addition, the IgG1 and IgG4 levels to Dpt were measured in these fractions to warrant the presence of high levels of these specific blocking antibodies. A low level of residual IgE antibody was detected in the chosen concentration of specific IgG fraction, suggesting a probable crossreactivity with the biotinylated anti-human IgE antibody used in ELISA, since no IgE reactivity was detected in this purified IgG fraction in immunoblot assays using another secondary antibody. 

The blocking activity of the allergen-specific IgG fraction on the IgE reactivity to Dpt allergen extract was then evaluated by inhibition ELISA. Allergen-specific IgG fraction was capable of reducing levels of IgE anti-Dpt, resulting in 35%–51% inhibition of IgE reactivity to Dpt in the three serum pools tested. Also, we verified that the presence of specific IgG1 or IgG4 or both subclasses together with specific IgE in the tested serum pools did not interfere with the IgE blocking capability of these specific IgG fractions. Considering that the inhibition phenomena of IgE binding seen for the specific IgG fractions include both IgG1 and IgG4 subclasses, it was not possible to attribute a more protective role to IgG4 compared to IgG1 in the design of the present study. However, as the IgG1/IgG4 ratio in the specific IgG fractions was higher than 1.0, it may be speculated that the specific IgG1 antibody could also play a protective role. Further studies should be conducted using absorption methods or purification of specific IgG1 or IgG4 fractions from patients that are single positive for each IgG subclass to evaluate separately the role of each subclass of specific IgG fraction. This blocking role of allergen-specific IgG antibodies has been recently investigated in mouse models through passive immunization with specific IgG antibodies for prevention and treatment of allergy to major birch and grass pollen allergens [39]. The authors showed that mice treated with anti-Phl p 1 IgG after sensitization with rPhl p 1 allergen had reduced Phl p 1-specific IgE levels in all time points tested. Also, inhibition percentages of IgE binding to Bet v 1 (23.8% to 57.4%) and Phl p 1 (30.8 to 63.3%) were found in the groups treated with the respective allergen-specific IgG antibodies [39]. In our study, when nonspecific IgG fraction was used, we also observed a decrease in Dpt-specific IgE levels, although with minor inhibition, which could be associated with the heterogeneity of the allergen composition of mite extracts [40, 41]. In this context, IgG antibodies could react with both allergenic and non-allergenic components present in the crude allergen extract, but only those IgE epitope-specific IgG antibodies belong to the true blocking IgG antibodies [41]. 

Altogether, our results showed that allergen-specific IgG antibodies purified from mite-allergic patient sera using available and standardized methodology are able to inhibit IgE reactivity to Dpt allergen extract. This approach reinforces that the intermittent measurement of serum allergen-specific IgG antibodies will be an important objective laboratorial parameter that will help specialists to follow their patients under allergen-specific immunotherapy.

## Figures and Tables

**Table 1:** Demographic and clinical characteristics of the study subjects.

Characteristics	Groups		*P* value
	Atopic	Nonatopic	
Number of subjects	36	15	—
Age (year)			
Mean ± SD	24.6 ± 5.8	28 ± 11.64	0.1725^a^
Sex (M : F)	13 : 23	2 : 13	0.1770^b^
Positive skin prick test (*n*, %)			
* Dermatophagoides pteronyssinus*	36 (100%)	0	<0.0001^b^
* Dermatophagoides farinae*	35 (97%)	0	<0.0001^b^
* Blomia tropicalis*	23 (64%)	0	<0.0001^b^
* Blatella germanica*	10 (28%)	0	0.0009^b^
* Periplaneta americana*	9 (25%)	0	0.0022^b^
* Alternaria alternata*	2 (6%)	0	0.4930^b^
* Felis domesticus*	19 (53%)	0	<0.0001^b^
* Canis familiaris *	14 (39%)	0	<0.0001^b^

^a^Student's *t*-test; ^b^Fisher's exact test (*P* < 0.05); SD: standard deviation.

**Table 2:** *Dermatophagoides  pteronyssinus*-specific IgE, IgG1, and IgG4 reactivity profile in serum pools of atopic (*n* = 5) and non-atopic (*n* = 5) subjects.

Dpt-specific antibody levels	Groups	
	Atopic	Nonatopic
	Dpt-specific serum pool	Nonspecific serum pool
IgE (mean EI ± SD)	4.28 ± 1.73	1.06 ± 0.54
IgG1 (mean EI ± SD)	6.43 ± 2.00	0.85 ± 0.17
IgG4 (mean EI ± SD)	4.51 ± 2.11	0.85 ± 0.19

Dpt: *Dermatophagoides pteronyssinus* allergen extract; EI: ELISA index; SD: standard deviation.

**Table 3:** *Dermatophagoides pteronyssinus*-specific IgE, IgG1, and IgG4 reactivity profile in three serum pools (I, II, and III) of atopic patients.

Dpt-specific antibody levels	Atopic serum pools		
	I	II	III
IgE (mean EI ± SD)	**4.46 ± 3.63**	**1.80 ± 0.70**	**4.20 ± 2.55**
IgG1 (mean EI ± SD)	**1.87 ± 0.60**	0.90 ± 0.06	**3.23 ± 2.06**
IgG4 (mean EI ± SD)	0.93 ± 0.22	**2.81 ± 2.17**	**4.35 ± 2.24**

Dpt: *Dermatophagoides pteronyssinus* allergen extract; EI: ELISA index; SD: standard deviation; positive values are represented in bold.
